# Association of gamma-glutamyl transferase variability with risk of venous thrombosis

**DOI:** 10.1038/s41598-023-34368-5

**Published:** 2023-05-06

**Authors:** Yoonkyung Chang, Heajung Lee, Tae-Jin Song

**Affiliations:** 1 Department of Neurology, Mokdong Hospital, Ewha Womans University College of Medicine, Seoul, Republic of Korea; 2 Department of Neurology, Seoul Hospital, Ewha Womans University College of Medicine, 260, Gonghang-Daero, Gangseo-Gu, Seoul, 07804 Republic of Korea

**Keywords:** Epidemiology, Infectious diseases

## Abstract

Gamma-glutamyl transferase (GGT) is a biomarker of inflammation, and is known to be associated with stroke and atrial fibrillation. Venous thromboembolism (VT), a not uncommon thrombotic disorder, shares similar mechanisms with other thrombotic disorders including these stroke and atrial fibrillation. Given these associations, we intended to investigate the potential association between variability in GGT and VT. The study included data from the National Health Insurance Service-Health Screening Cohort, comprising 1,085,105 participants with health examinations 3 or more times from 2003 to 2008. Variability indexes were the coefficient of variation, standard deviation, and variability independent of the mean. The occurrence of venous thromboembolism (VT) was defined with more than one claim of the following ICD-10 codes: deep VT (I80.2–80.3), pulmonary thromboembolism (I26), intraabdominal venous thrombosis (I81, I82.2, I82.3), or other VT (I82.8, I82.9). To determine the relationship of quartiles of GGT with incident VT risk, Kaplan–Meier survival curve and logrank test were used. Cox’s proportional hazard regression was used to investigate the risk of VT occurrence by GGT quartile (Q1–Q4). A total of 1,085,105 subjects were incorporated in the analysis, and the average follow-up was 12.4 years (interquartile range 12.2–12.6). VT occurred in 11,769 (1.08%) patients. The GGT level was measured 5,707,768 times in this stud. Multivariable analysis showed that GGT variability were positively associated with the occurrence of VT. Compared to the Q1, the Q4 showed an adjusted HR of 1.15 (95% CI 1.09–1.21, *p* < 0.001) when using coefficient of variation, 1.24 (95% CI 1.17–1.31, *p* < 0.001) when using standard deviation, and 1.10 (95% CI 1.05–1.16, *p* < 0.001) when using variability independent of the mean. Increased variability of GGT may be related to an increased risk of VT. Maintaining a stable GGT level would be beneficial in reducing the risk of VT.

## Introduction

Gamma-glutamyl transferase (GGT) is a representative biomarker of liver diseases. Recent studies have reported other diseases related to GGT, particularly end-stage renal disease, cardiovascular and cerebrovascular disease, leading to mortality^1–6^. In order to determine the association between a disease and a specific biomarker, multiple measurements are more reliable than one measurement. Also, since variability itself is an important biomarker, the variability of the biomarker must be measured repeatedly. Moreover, variability in biomarkers such as blood pressure, blood glucose levels, and lipid profiles can affect the progression of diseases. Previous studies have shown that the variability of GGT is closely linked to the risk of acute coronary syndrome, heart failure, and stroke^7,8^.

Venous thrombosis (VT) is not uncommon and has a high worldwide disease burden, resulting in mortality in certain cases^9^. The incidence of VT is increasing globally due to the aging society^10^. There are known risk factors for VT, such as cancer, fracture, renal failure, chronic inflammatory disorders, antiphospholipid antibody syndrome, and obesity^10^. Nevertheless, there are needs for preventable and correctable risk factors for VT.

In prior studies, increased blood GGT was related with venous thrombosis and atrial fibrillation, which is closely related to venous thrombosis^11–13^. These reports provided evidence that GGT variability may be involved in VT. In addition, research has shown a strong association between variations in GGT and other metabolic parameters with inflammation-related metabolism and dysregulation of hematologic homeostasis. These factors could potentially contribute to the development of VT^3,14–17^. However, to date, there is a scarcity of information regarding the link between VT and prolonged changes in GGT levels. We hypothesized that GGT variability is related with VT occurrence. The purpose of this study was to examine the relationship between GGT variability and the occurrence of VT in a population-based nationwide cohort database.

## Results

A total of 1,085,105 subjects were entered in the study (Average follow-up 12.4 years, interquartile range 12.2–12.6). VT occurred in 11,769 (1.08%) patients: deep vein thrombosis (4710 (0.43%)), pulmonary thromboembolism (3109 (0.29%)), intraabdominal thrombosis (5214 (0.48%)), and other VT (4793 (0.44%)). The GGT was checked 5,707,768 times (number of participants for 3 times: 114,630, 4 times: 133,486, 5 times: 192,000, 6 times: 644,989). The comparative analysis results on the study subjects according to quartile (Q1, lowest–Q4, highest) of coefficient of variation are presented in Table 1. Participants with Q4 were more commonly older men, and had comorbid diseases more frequently compared to Q1.

**Table 1:** Baseline characteristics of subjects according to GGT variability.

Variable	Total	Q1	Q2	Q3	Q4	*p* value
Number of participants (%)	1,085,105	271,327 (25.0)	271,225 (25.0)	271,281 (25.0)	271,272 (25.0)	
Age, years	43.81 ± 10.14	43.82 ± 10.26	43.61 ± 9.93	43.57 ± 9.94	44.24 ± 10.39	< .001
Sex						< .001
Male	835,894 (77.0)	198,578 (73.2)	208,673 (76.9)	213,642 (78.8)	215,001 (79.3)	
Female	249,211 (23.0)	72,749 (26.8)	62,552 (23.1)	57,639 (21.3)	56,271 (20.7)	
Body mass index (kg/m^2^)	23.77 ± 3.01	23.27 ± 2.97	23.67 ± 2.99	23.97 ± 3.00	24.15 ± 3.02	< .001
Household income						< .001
Q1, lowest	158,311 (14.6)	40,155 (14.8)	37,142 (13.7)	37,599 (13.9)	43,415 (16.0)	
Q2	338,611 (31.2)	84,556 (31.2)	82,123 (30.3)	83,392 (30.7)	88,540 (32.6)	
Q3	396,077 (36.5)	97,321 (35.9)	101,065 (37.3)	101,112 (37.3)	96,579 (35.6)	
Q4, highest	192,106 (17.7)	49,295 (18.2)	50,895 (18.8)	49,178 (18.1)	42,738 (15.8)	
Smoking status						< .001
Never	557,395 (51.4)	151,281 (55.8)	140,774 (51.9)	134,263 (49.5)	131,077 (48.3)	
Former	161,055 (14.8)	37,622 (13.9)	40,567 (15.0)	41,377 (15.3)	41,489 (15.3)	
Current	366,655 (33.8)	82,424 (30.4)	89,884 (33.1)	95,641 (35.3)	98,706 (36.4)	
Alcohol consumption (days/week)						< .001
None	675,751 (62.3)	186,121 (68.6)	173,485 (64.0)	162,927 (60.1)	153,218 (56.5)	
1–4	390,190 (36.0)	82,160 (30.3)	93,987 (34.7)	103,550 (38.2)	110,493 (40.7)	
≥ 5	19,164 (1.8)	3046 (1.1)	3753 (1.4)	4804 (1.8)	7561 (2.8)	
Regular physical activity (days/week)						< .001
None	467,512 (43.1)	119,872 (44.2)	116,375 (42.9)	115,182 (42.5)	116,083 (42.8)	
1–4	549,438 (50.6)	135,189 (49.8)	138,209 (51.0)	139,124 (51.3)	136,916 (50.5)	
≥ 5	68,155 (6.3)	16,266 (6.0)	16,641 (6.1)	16,975 (6.3)	18,273 (6.7)	
Comorbidities						
Hypertension	213,961 (19.7)	44,511 (16.4)	49,163 (18.1)	54,534 (20.1)	65,753 (24.2)	< .001
Diabetes mellitus	107,957 (10.0)	20,835 (7.7)	23,362 (8.6)	27,367 (10.1)	36,393 (13.4)	< .001
Dyslipidemia	210,802 (19.4)	42,505 (15.7)	48,005 (17.7)	54,219 (20.0)	66,073 (24.4)	< .001
Stroke	10,757 (1.0)	2176 (0.8)	2283 (0.8)	2559 (0.9)	3739 (1.4)	< .001
Atrial fibrillation	4135 (0.4)	812 (0.3)	845 (0.3)	937 (0.4)	1541 (0.6)	< .001
Renal disease	12,792 (1.2)	2489 (0.9)	2750 (1.0)	3194 (1.2)	4359 (1.6)	< .001
Cancer	23,959 (2.2)	5111 (1.9)	5214 (1.9)	5595 (2.1)	8039 (3.0)	< .001
Antiphospholipid syndrome	3983 (0.4)	806 (0.3)	868 (0.3)	935 (0.3)	1374 (0.5)	< .001
Osteoporotic fracture	15,343 (1.4)	3662 (1.4)	3569 (1.3)	3721 (1.4)	4391 (1.6)	< .001
Aspartate aminotransferase (U/L)	25.14 ± 12.41	22.94 ± 9.08	24.02 ± 10.24	25.41 ± 15.77	29.82 ± 32.65	< .0001
Alanine aminotransferase (U/L)	26.12 ± 17.42	22.35 ± 13.79	24.83 ± 15.85	27.60 ± 19.91	34.10 ± 41.54	< .0001
Mean gamma-glutamyl transferase (U/L)	37.97 ± 38.06	27.10 ± 23.42	32.21 ± 28.81	38.54 ± 35.20	54.02 ± 52.60	< .001
GGT variability						
CV (%)	26.87 ± 16.17	11.84 ± 3.21	19.84 ± 2.04	27.84 ± 2.77	47.95 ± 17.40	< .001
SD	12.01 ± 20.72	3.26 ± 3.10	6.42 ± 5.88	10.79 ± 10.11	27.59 ± 34.92	< .001
VIM (%)	10.52 ± 5.86	4.93 ± 1.39	8.08 ± 1.14	11.07 ± 1.61	17.99 ± 6.20	< .001

*p* value by Chi-square test. Data are expressed as the mean ± SD, or n (%).

Q; quartile, GGT; Gamma-glutamyl Transferase, CV, coefficient of variation; SD, standard deviation; VIM, variability independent of the mean.

**Figure 1:**
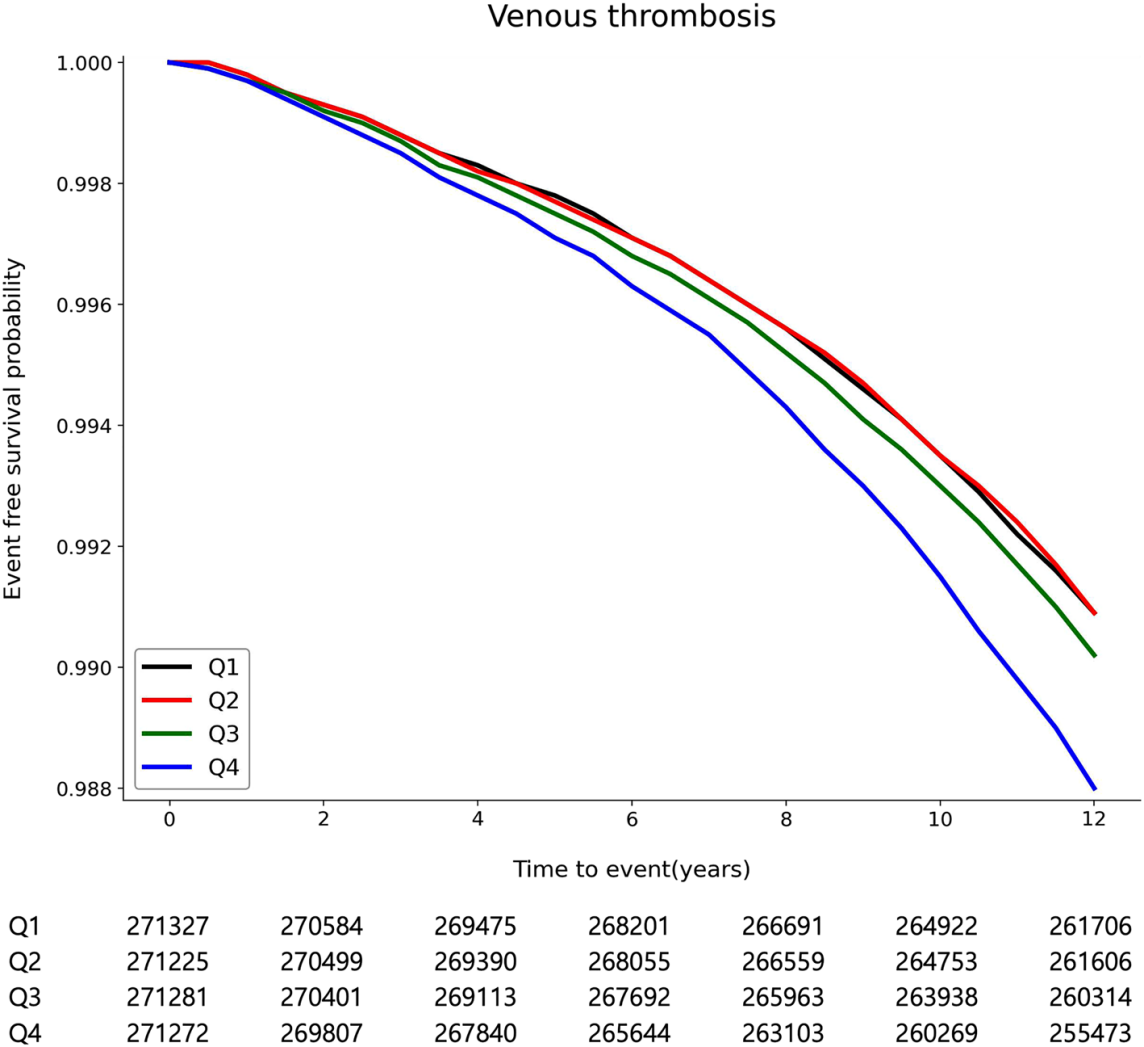
Kaplan–Meier survival curves for occurrence of VT according to GGT variability.

Figure 1 shows Kaplan–Meier survival curve for VT occurrence according to GGT variability. The risk for VT occurrence was found to increase significantly in Q4 (*p* < 0.001). Moreover, in multivariable analysis, variability of GGT were positively associated with the occurrence of VT. The Q4 showed an adjusted hazard ratio (HR) of 1.15 (95% CI 1.09–1.21, *p* for trend < 0.001, *p* < 0.001) when using coefficient of variation, 1.24 (95% CI 1.17–1.31, *p* for trend < 0.001, *p* < 0.001) when using standard deviation (SD), and 1.10 (95% CI 1.05–1.16, p for trend < 0.001, p < 0.001) when using variability independent of the mean (Table 2, Supplementary Table 1). Moreover, the association remained constant after adjustment of mean GGT level (coefficient of variation; adjusted HR 1.08, 95% CI 1.03–1.14, *p* for trend < 0.001, *p* = 0.004, SD; adjusted HR 1.09, 95% CI 1.03–1.16, *p* for trend = 0.010, *p* = 0.005, variation independent of the mean; adjusted HR 1.07, 95% CI 1.02–1.13, *p* for trend = 0.017, *p* = 0.007, Table 2). Regarding deciles of GGT variability, increased GGT level variability was related with occurrence of VT (coefficient of variation; adjusted HR 1.24, 95% CI 1.15–1.34, *p* for trend < 0.001, *p* < 0.001, SD; adjusted HR 1.35, 95% CI 1.24–1.48, *p* for trend < 0.001, *p* < 0.001, variation independent of the mean; adjusted HR 1.18, 95% CI 1.09–1.28, *p* for trend = 0.001, *p* < 0.001, Supplementary Table 2). The correlation was significant even when the VT occurrence was re-defined as 1 year after the index date (adjusted HR 1.14, 95% CI 1.09–1.20, *p* < 0.001, Supplementary Tables 3, 4).

**Table 2:** The risk for occurrence of venous thrombosis according to quartiles of GGT variability.

	Number of participants	Number of events	Event rate (%) (95% CI)	Person-years	Incidence rate (per 1000 person-years)	Multivariable model (1)			Multivariable model (2)		
						Adjusted HR (95% CI)	*p* value	*p* value for trend	Adjusted HR (95% CI)	*p* value	*p* value for trend
CV								< .001			< .001
Q1	271,327	2683	0.99 (0.95, 1.03)	3,319,950.62	0.81	1 (reference)			1 (reference)		
Q2	271,225	2694	0.99 (0.96, 1.03)	3,318,230.12	0.81	1.01 (0.96, 1.07)	0.730		1.00 (0.95, 1.06)	0.937	
Q3	271,281	2891	1.07 (1.03, 1.10)	3,310,959.00	0.87	1.06 (1.00, 1.11)	0.044		1.04 (0.98, 1.09)	0.192	
Q4	271,272	3501	1.29 (1.25, 1.33)	3,280,957.92	1.07	1.15 (1.09, 1.21)	< .001		1.08 (1.03, 1.14)	0.004	
SD								< .001			0.010
Q1	271,920	2435	0.90 (0.86, 0.93)	3,336,298.93	0.73	1 (reference)			1 (reference)		
Q2	270,447	2674	0.99 (0.95, 1.03)	3,308,877.37	0.81	1.01 (0.95, 1.06)	0.825		1.00 (0.95, 1.06)	0.988	
Q3	271,459	2962	1.09 (1.05, 1.13)	3,311,104.43	0.90	1.05 (0.99, 1.11)	0.086		1.03 (0.97, 1.09)	0.373	
Q4	271,279	3698	1.36 (1.32, 1.41)	3,273,816.94	1.13	1.24 (1.17, 1.31)	< .001		1.09 (1.03, 1.16)	0.005	
VIM								0.001			0.017
Q1	271,281	2764	1.02 (0.98, 1.06)	3,315,439.52	0.83	1 (reference)			1 (reference)		
Q2	271,275	2764	1.02 (0.98, 1.06)	3,316,312.42	0.83	1.03 (0.98, 1.09)	0.291		1.03 (0.97, 1.08)	0.353	
Q3	271,276	2943	1.08 (1.05, 1.12)	3,310,317.05	0.89	1.08 (1.03, 1.14)	0.003		1.07 (1.02, 1.13)	0.009	
Q4	271,273	3298	1.22 (1.17, 1.26)	3,288,028.68	1.00	1.10 (1.05, 1.16)	< .001		1.07 (1.02, 1.13)	0.007	

Multivariable model (1) was adjusted for sex, age, body mass index, income levels, smoking, alcohol consumption, regular physical activity, hypertension, diabetes mellitus, dyslipidemia, stroke, atrial fibrillation, renal disease, cancer, antiphospholipid syndrome, osteoporotic fracture, aspartate aminotransferase, and alanine aminotransferase.

Multivariable model (2) was adjusted for sex, age, body mass index, income levels, smoking, alcohol consumption, regular physical activity, hypertension, diabetes mellitus, dyslipidemia, stroke, atrial fibrillation, renal disease, cancer, antiphospholipid syndrome, osteoporotic fracture, aspartate aminotransferase, alanine aminotransferase, and mean GGT.

GGT: Gamma-glutamyl Transferase, CI: confidence interval, HR: hazard ratio, Q: quartile, CV: coefficient of variation, SD: standard deviation, VIM: variability independent of the mean.

In subgroup analysis, the Q4 of GGT variability was positively related with risk of deep VT, pulmonary thromboembolism, intraabdominal thrombosis, and other VT compared with Q1 (Supplementary Tables 5–8, Supplementary Figs. 1–4).

## Discussion

The main results of our study demonstrated that variability of GGT was related to an increased risk of VT. Moreover, this finding was consistent regardless of the type of VT (deep VT, pulmonary thromboembolism, intraabdominal thrombosis, and other VT).

Previous studies have shown relationships of stroke and cardiovascular disease with GGT^18–20^. In a meta-analysis, GGT was related with stroke, cardiovascular, and all-cause mortality^18,19^. High GGT levels had a positive linking with increased stroke risk, and the highest GGT quartile had about 1.5 times higher cardiovascular and all-cause mortality risk than the lowest quartile^18,19^. In another study of 698,937 diabetic patients without known cardiovascular disease, chronic liver disease, and heavy alcohol consumption, the risk of stroke and death increased by 6% and 23%, respectively, in the group with increased GGT variability^21^. In a general population-based study that investigated GGT and hospitalization for heart failure, 1.16% of events occurred during 8.4 years of follow-up, and the risk of hospitalization was high in the group with high GGT variability, with an HR of 1.22^8^. Our study confirmed that the risk of venous thrombosis increased when GGT variability was high. It can be inferred that GGT oscillation is related to the occurrence of thrombotic disease due to homeostasis failure as well as an increase in GGT.

Our study demonstrated the relationship of GGT variability and VT. Moreover, the relationship was consistent in the subgroup analysis, especially in deep VT and pulmonary thromboembolism. While PTE is the one of the diseases with high mortality, our study suggested additional information on VT, especially deep VT and pulmonary thromboembolism occurrence.

Although our study does not explain the mechanism, there are some possible hypotheses on the results of our study. GGT is involved in glutathione homeostasis^22^. Glutathione is an anti-oxidant synthesized by glutamate-cysteine ligase and glutathione synthase^23^. Elevated reactive oxygen species (ROS) can cause oxidative damage to cells^24^, and glutathione has a protective effect on ROS^25^. GGT is involved in degrading extracellular glutathione and providing cysteine during synthesis of glutathione^26^. GGT elevation promotes ROS generation and causes oxidative stress^27^, which seems to be involved in the occurrence of cardiovascular disease. The development of venous thrombosis is also affected by ROS, which influence the formation and degradation of thrombus through the coagulation pathway, fibrinolysis, and effector cells including red blood cells and platelets^28,29^. Although the exact mechanism by which GGT variability causes VT is not known, it is presumed that GGT may affect the occurrence of VT as a mechanism similar to how variability in blood pressure or blood sugar adversely affects arteriosclerosis^30,31^. Blood pressure variability affects progression of atherosclerosis by increasing inflammation, mechanical stimulation of vessels, and vascular smooth muscle cell dysfunction^30^. Considering that GGT induction is increased by oxidative stress^32^, an increase in GGT variability may indicate recurrent oxidative stress.

This study had several limitations. First, there is a possibility of other confounding factors such as coagulation tests including d-dimer and C-reactive protein, which were unavailable in our dataset. Second, the study population are Korean, and the results could not be applied to other ethnicities. Third, the retrospective observational design of our study does not allow us to establish a clear causal connection and presents challenges in identifying the exact cause of GGT variability. Although our study goal was to confirm the association of VT with a fixed estimate of the GGT variability for the prior 6 years before index date, we did not consider GGT variability may change in the follow-up periods. Fourth, cerebral VT, which mainly occurs in young women, was excluded in our study because our dataset consists of individuals older than 40 years. Fifth, this study may exhibit selection bias as it exclusively includes participants who have undergone health screening examinations, potentially resulting in a sample comprised solely of healthy individuals. Lastly, diagnostic accuracy of VT with ICD-10 codes in the National Health Insurance Service-National Health Screening (NHIS-HEALS) could not be clearly presented.

Despite the limitations, this study has some strengths. This study utilized a nationally representative data over a significant period to examine the impact of GGT variability on VT. Our findings provide compelling evidence confirming the importance of retaining a stable GGT level as a preventive measure against VT.

## Conclusion

Increased GGT variability may be linked with increased risk of VT. Maintaining stable GGT level would be helpful for reducing the risk of VT. Further studies on the mechanisms responsible for the association between GGT variability and VT development are needed.

## Methods

### Data source

This study utilized the NHIS-HEALS cohort database from Korea. The NHIS is a government-controlled insurance provider that covers 97% of Koreans. The remaining are covered by the Medical Aid program, which is also administered by the government^33–35^. Annual standardized health screenings are provided by NHIS. The cohort used in this study comprised randomly selected individuals between 40 and 79 years of age, who had done at least three health screenings (Dataset number: NIHS-2021-01-715)^36–38^. The NHIS-HEALS cohort database used in this study includes demographic data, socioeconomic status, and health screening information, as well as a claims database that contains information on diagnosis, prescription, and treatment methods. The health screening process involved measurements of weight, height, laboratory results, and lifestyle questionnaire such as smoking and alcohol history. The NHIS-HEALS does not have any role in this study. The study analysis was approved by the Institutional Review Board of Ewha Womans University College of Medicine (2021-12-038), and consent was waived. This study is performed in accordance with the Declaration of Helsinki.

### Study population and variables

**Figure 2:**
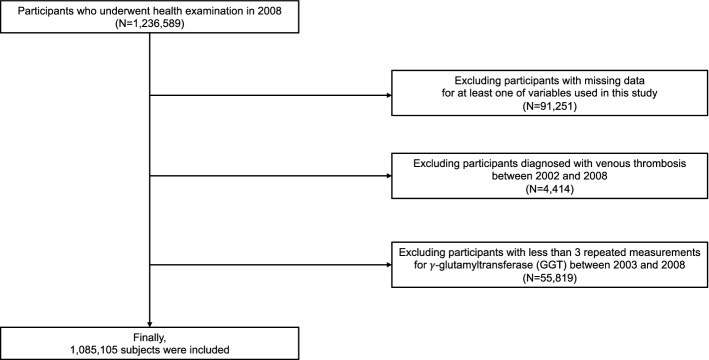
Flow chart of study subjects.

The participants with health examination 3 times or more between 2003 and 2008 were included from the NIHS-HEALS database (n = 1,236,589). Participants with missing data for analysis (n = 91,251) were excluded. Furtherer, participants with a previous history of VT (n = 4414) were excluded. Finally, 1,085,105 participants were investigated in this study (Fig. 2). A detailed description of the definition of variables can be found in the supplementary methods (Supplementary methods).

### Definition of GGT variability

The definition of GGT variability used in this study refers to the intraindividual variability of GGT values obtained from each examination conducted during the six years preceding the index year (2009). Three variability indexes examined were coefficient of variation, SD, and variability independent of the mean. The formular for variability independent of the mean was 100 × SD/Mean^beta^, where beta is the regression coefficient based on the natural logarithm of the standard deviation over the natural logarithm of the mean^39^.

### Study outcomes

The primary outcome of the study was VT occurrence, which was defined as the presence of more than one claims with diagnostic codes corresponding to any of the following ICD-10 codes: [deep VT (I80.2–80.3), pulmonary thromboembolism (I26, I26.0, I26.9), intraabdominal thrombosis (I81, I82, I82.2, I82.3), other VT (I82.8, I82.9)] with code for anticoagulants and antiplatelet, based on a previous study^40^. The follow-up period was from the index date to VT occurrence, death, or December 2020, whichever came first.

### Statistical analysis

The study used the Chi-square test and analysis of variance test to compare the demographics of different groups. All GGT variability was found to have a positive linear association, confirmed by restricted cubic splines^41^. Kaplan–Meier survival curve along with logrank test were used to access the association of quartiles of GGT with incident VT risk. The study calculated the incidence of VT as the number of cases divided by the sum of person-years. Cox's proportional hazard regression was used to determine the risk of VT occurrence by GGT quartile, and the hazard ratio (HR) and 95% confidence interval (CI) were determined. A multivariable regression model with adjustments for several factors, including age, sex, body mass index, domestic income, regular physical activity, alcohol drinking, smoking status, and comorbidities (diabetes mellitus, hypertension, dyslipidemia, renal disease, stroke, atrial fibrillation, cancer, antiphospholipid antibody syndrome, and osteoporotic fracture), aspartate aminotransferase, and alanine aminotransferase was constructed. Shoenfeld's residuals were performed, and no departure from the proportional hazards’ assumption was detected. Subgroup analysis analyses were performed to determine the risk of each kind of VT (deep VT, pulmonary thromboembolism, intraabdominal thrombosis, and other VT). Sensitivity analysis was conducted by adjusting for (1) mean GGT level in multivariable analysis, (2) coefficient of variation, SD, and variability independent of the mean according to decile instead of quartile, and (3) excluding participants with VT within 1 year from the index date to minimize the possibility of reverse causality. Statistical Analysis System software (SAS version 9.2, SAS Institute, Cary, NC) was used in statistical evaluations. A *p* value < 0.05 was considered statistically significant.

### Ethical approval statement

The Institutional Review Board of Ewha Womans University College of Medicine (2021-12-038) approved the analysis and provided a consent waiver as the data were anonymized and freely accessible by the NHIS for study purposes.

## Supplementary Information

Supplementary Information.

## Data Availability

The data used in this study are available in the National Health Insurance Service-National Health Screening Cohort (NHIS-HEALS) database, but restrictions apply to public availability of these data used under license for the current study. Requests for access to the NHIS data can be made through the National Health Insurance Sharing Service homepage (http://nhiss.nhis.or.kr/bd/ab/bdaba021eng.do). For access to the database, a completed application form, research proposal, and application for approval from the institutional review board should be submitted to the inquiry committee of research support in the NHIS for review.

## References

[1] Ndrepepa G, Colleran R, Kastrati A (2018). Gamma-glutamyl transferase and the risk of atherosclerosis and coronary heart disease. Clin. Chim. Acta.

[2] Lee DH (2006). Serum gamma-glutamyltransferase predicts non-fatal myocardial infarction and fatal coronary heart disease among 28,838 middle-aged men and women. Eur. Heart J..

[3] Lee DY (2020). Gamma-glutamyl transferase variability can predict the development of end-stage of renal disease: A nationwide population-based study. Sci. Rep..

[4] Kim YG (2020). Association of gamma-glutamyl transferase with subclinical coronary atherosclerosis and cardiac outcomes in non-alcoholics. Sci. Rep..

[5] Ruban A (2020). Liver enzymes and risk of stroke: The atherosclerosis risk in communities (ARIC) study. J. Stroke.

[6] Yang W, Kang DW, Lee SH (2020). Effects of gamma-glutamyl transferase on stroke occurrence mediated by atrial fibrillation. J. Clin. Neurol..

[7] Chung HS (2019). γ-Glutamyltransferase variability and the risk of mortality, myocardial infarction, and stroke: A nationwide population-based cohort study. J. Clin. Med..

[8] Hong SH (2020). Gamma-glutamyl transferase variability and the risk of hospitalisation for heart failure. Heart.

[9] Tagalakis V, Patenaude V, Kahn SR, Suissa S (2013). Incidence of and mortality from venous thromboembolism in a real-world population: The Q-VTE Study Cohort. Am. J. Med..

[10] Khan F, Tritschler T, Kahn SR, Rodger MA (2021). Venous thromboembolism. Lancet.

[11] Folsom AR (2014). Elevated hepatic enzymes and incidence of venous thromboembolism: A prospective study. Ann. Epidemiol..

[12] Lee SR, Choi EK, Han KD, Cha MJ, Oh S (2017). Association between γ-glutamyltransferase level and incidence of atrial fibrillation: A nationwide population-based study. Int. J. Cardiol..

[13] Lutsey PL (2018). Atrial fibrillation and venous thromboembolism: evidence of bidirectionality in the Atherosclerosis Risk in Communities Study. J. Thromb. Haemost..

[14] Williams DP (2019). Heart rate variability and inflammation: A meta-analysis of human studies. Brain Behav. Immun..

[15] Hoffman RP, Dye AS, Huang H, Bauer JA (2016). Glycemic variability predicts inflammation in adolescents with type 1 diabetes. J. Pediatr. Endocrinol. Metab..

[16] Kim KI (2008). Association between blood pressure variability and inflammatory marker in hypertensive patients. Circ. J..

[17] Cho EJ, Han K, Lee SP, Shin DW, Yu SJ (2020). Liver enzyme variability and risk of heart disease and mortality: A nationwide population-based study. Liver Int..

[18] Zhang XW (2015). Association between gamma-glutamyltransferase level and risk of stroke: A systematic review and meta-analysis of prospective studies. J. Stroke Cerebrovasc. Dis..

[19] Du G, Song Z, Zhang Q (2013). Gamma-glutamyltransferase is associated with cardiovascular and all-cause mortality: A meta-analysis of prospective cohort studies. Prev. Med..

[20] Yao T (2019). Association between serum gamma-glutamyl transferase and intracranial arterial calcification in acute ischemic stroke subjects. Sci. Rep..

[21] Lee DY (2020). Prognostic value of long-term gamma-glutamyl transferase variability in individuals with diabetes: A nationwide population-based study. Sci. Rep..

[22] Whitfield JB (2001). Gamma glutamyl transferase. Crit. Rev. Clin. Lab. Sci..

[23] Lu SC (2013). Glutathione synthesis. Biochim. Biophys. Acta.

[24] Pizzino G (2017). Oxidative stress: Harms and benefits for human health. Oxid. Med. Cell Longev..

[25] Fernández-Checa JC (1997). GSH transport in mitochondria: Defense against TNF-induced oxidative stress and alcohol-induced defect. Am. J. Physiol..

[26] Zhang H, Forman HJ, Choi J (2005). Gamma-glutamyl transpeptidase in glutathione biosynthesis. Methods Enzymol..

[27] Lee DH, Jacobs DR (2005). Association between serum gamma-glutamyltransferase and C-reactive protein. Atherosclerosis.

[28] Gutmann C, Siow R, Gwozdz AM, Saha P, Smith A (2020). Reactive oxygen species in venous thrombosis. Int. J. Mol. Sci..

[29] Wang Q, Zennadi R (2020). Oxidative stress and thrombosis during aging: The roles of oxidative stress in RBCs in venous thrombosis. Int. J. Mol. Sci..

[30] Liu Y, Luo X, Jia H, Yu B (2022). The effect of blood pressure variability on coronary atherosclerosis plaques. Front. Cardiovasc. Med..

[31] Mo Y (2013). Glycemic variability is associated with subclinical atherosclerosis in Chinese type 2 diabetic patients. Cardiovasc. Diabetol..

[32] Kugelman A (1994). gamma-Glutamyl transpeptidase is increased by oxidative stress in rat alveolar L2 epithelial cells. Am. J. Respir. Cell Mol. Biol..

[33] Chang Y, Woo HG, Park J, Lee JS, Song TJ (2020). Improved oral hygiene care is associated with decreased risk of occurrence for atrial fibrillation and heart failure: A nationwide population-based cohort study. Eur. J. Prev. Cardiol..

[34] Chang Y, Lee JS, Lee KJ, Woo HG, Song TJ (2020). Improved oral hygiene is associated with decreased risk of new-onset diabetes: A nationwide population-based cohort study. Diabetologia.

[35] Park, J.-H. *et al.* Gradual, but not sudden, dose-dependent increase of ONJ risk with bisphosphonate exposure: A nationwide cohort study in women with osteoporosis. **12** (2021). 10.3389/fendo.2021.774820 PMC8695600 34956086

[36] Park MS, Jeon J, Song TJ, Kim J (2022). Association of periodontitis with microvascular complications of diabetes mellitus: A nationwide cohort study. J Diabetes Compl..

[37] Chang Y (2021). Improved oral hygiene care and chronic kidney disease occurrence: A nationwide population-based retrospective cohort study. Medicine (Baltimore).

[38] Seong SC (2017). Cohort profile: The National Health Insurance Service-National Health Screening Cohort (NHIS-HEALS) in Korea. BMJ Open.

[39] Tian X (2021). Visit-to-visit variability of serum uric acid measurements and the risk of all-cause mortality in the general population. Arthritis Res. Ther..

[40] Jang MJ, Bang S-M, Oh D (2011). Incidence of venous thromboembolism in Korea: From the health insurance review and assessment service database. J. Thromb. Haemost..

[41] Desquilbet L, Mariotti F (2010). Dose-response analyses using restricted cubic spline functions in public health research. Stat Med.

