# Supplementary material for: Association of gamma-glutamyl transferase variability with risk of venous thrombosis
Source: Sci Rep. 2023 May 6;13:7402. doi: 10.1038/s41598-023-34368-5 (PMC10164162; doi:10.1038/s41598-023-34368-5)
Supplement: Supplementary file 1 — Supplementary Information. [file 41598_2023_34368_MOESM1_ESM.pdf]

Title: Association of gamma-glutamyl transferase variability with risk of venous thrombosis

Supplementary methods

Definition of variables

The index date was the date of the health examination. At the index date, we collected baseline characteristics including age, sex, household income, and body mass index. The participants' smoking habits (categorized as current, former, or never), alcohol consumption (reported as average frequency per week: <1 time, 1–2 times, 3–4 times, or ≥5 times), and physical activity (reported as average frequency per week: <1 day, 1–4 days, or ≥5 days) were obtained through a self-report questionnaire on their lifestyles, as part of the health check-up program. Additionally, their household income was classified into quartiles based on their national health insurance payment history. We also identified comorbidities between January 2002 and the index date, including hypertension, diabetes mellitus, dyslipidemia, stroke, atrial fibrillation, cancer, and renal disease. Hypertension was defined as meeting at least one of the following criteria: 1) having at least one claim with a diagnostic code (International Classification of Diseases, Tenth Revision (ICD)-10 I10–15) and receiving an antihypertensive agent prescription, 2) having two or more claims of diagnostic codes (ICD-10 E11–14), 3) having a systolic/diastolic blood pressure ≥140/90 mmHg, or 4) self-reporting hypertension in the questionnaire. Diabetes mellitus was defined as meeting at least one of the following criteria: 1) having at least one claim of diagnostic codes (ICD-10 E11–14) and receiving an antidiabetic agent prescription, 2) having two or more claims of diagnostic codes

(ICD-10 E11–14), 3) having a fasting serum glucose level ≥ 7.0 mmol/L, or 4) self-reporting diabetes mellitus in the questionnaire. Dyslipidemia was defined as meeting at least one of the following criteria: 1) having at least one claim of diagnostic codes (ICD-10 E78) and receiving a dyslipidemia-related agent prescription, 2) having two or more claims of diagnostic codes (ICD-10 E78), or 3) having a total cholesterol ≥ 240 mg/dL. Stroke was defined as having two or more claims of diagnostic code (ICD-10 I60–64) with brain CT/MRI and hospital admission. Atrial fibrillation was defined as having two or more claims of diagnostic code (ICD-10 I48). Cancer was defined as having one admission or at least three outpatient claims of a diagnostic code (ICD-10 C00–97) with specific registration code of 'V027' or 'V193–4.' Renal disease was defined as having two or more claims of diagnostic codes (ICD 10 N17-19, I12-13, E082, E102, E112, E132) or an estimated glomerular filtration rate less than 60 mL/min/1.73m^2.[1-6] Antiphospholipid antibody syndrome was defined as two or more claims of diagnostic codes (ICD-10 D68.6) and a rare intractable disease program (V253). Osteoporotic fracture was defined as the presence of one of the following ICD-10 codes: 1) vertebral fracture (S22.0, S22.1, S32.0, S32.7, T08, M48.4, M48.5, M49.5), 2) hip fracture (S72.0, S72.1), distal radius fracture (S52.5, S52.6), and humerus fracture (S42.2, S42.3), which were validated and based on the Korean society of Bone and Mineral Research.[7]

References

1 Song, T. J., Kim, J. W. & Kim, J. Oral health and changes in lipid profile: A nationwide cohort study. *J Clin Periodontol* **47**, 1437-1445, doi:10.1111/jcpe.13373 (2020).

2 Woo, H. G., Chang, Y., Lee, J. S. & Song, T. J. Association of Tooth Loss with New-

Onset Parkinson's Disease: A Nationwide Population-Based Cohort Study. *Parkinsons Dis* **2020**, 4760512, doi:10.1155/2020/4760512 (2020).

3 Chang, Y., Woo, H. G., Lee, J. S. & Song, T. J. Better oral hygiene is associated with lower risk of stroke. *J Periodontol* **92**, 87-94, doi:10.1002/JPER.20-0053 (2021).

4 Lee, K. *et al.* Oral health and gastrointestinal cancer: A nationwide cohort study. *J Clin Periodontol* **47**, 796-808, doi:10.1111/jcpe.13304 (2020).

5 Kim, J., Kim, H. J., Jeon, J. & Song, T. J. Association between oral health and cardiovascular outcomes in patients with hypertension: a nationwide cohort study. *J Hypertens* **40**, 374-381, doi:10.1097/HJH.0000000000003022 (2022).

6 Song, T. J., Chang, Y., Jeon, J. & Kim, J. Oral health and longitudinal changes in fasting glucose levels: A nationwide cohort study. *PLoS One* **16**, e0253769, doi:10.1371/journal.pone.0253769 (2021).

7 Ahn, S. H. *et al.* Osteoporosis and Osteoporotic Fracture Fact Sheet in Korea. *J Bone Metab* **27**, 281-290, doi:10.11005/jbm.2020.27.4.281 (2020).

Supplementary Table 1. Risk factors for the occurrence of venous thrombosis

			Multivariable model (1)		Multivariable model (2)	
Variable	Crude HR (95% CI)	p-value	Adjusted HR (95% CI)	p-value	Adjusted HR (95% CI)	p-value
Age, years	1.08 (1.07, 1.08)	<.001	1.06 (1.06, 1.07)	<.001	1.06 (1.06, 1.07)	<.001
Sex						
Male	1 (reference)		1 (reference)		1 (reference)	
Female	1.18 (1.13, 1.23)	<.001	1.01 (0.96, 1.06)	0.800	1.05 (1.00, 1.11)	0.042
Body mass index (kg/m2)	1.07 (1.06, 1.07)	<.001	1.04 (1.04, 1.05)	<.001	1.04 (1.04, 1.05)	<.001
Household income						
Q1, lowest	1 (reference)		1 (reference)		1 (reference)	
Q2	0.60 (0.57, 0.63)	<.001	1.00 (0.95, 1.06)	0.905	1.00 (0.95, 1.06)	0.901
Q3	0.49 (0.47, 0.52)	<.001	0.89 (0.84, 0.93)	<.001	0.89 (0.84, 0.93)	<.001
Q4, highest	0.52 (0.49, 0.55)	<.001	0.72 (0.67, 0.76)	<.001	0.72 (0.68, 0.77)	<.001
Smoking status						
Never	1 (reference)		1 (reference)		1 (reference)	
Former	0.87 (0.82, 0.92)	<.001	1.01 (0.95, 1.07)	0.873	1.01 (0.95, 1.07)	0.879
Current	0.77 (0.74, 0.81)	<.001	1.10 (1.05, 1.15)	<.001	1.08 (1.03, 1.13)	0.003
Alcohol consumption (days/week)						
None	1 (reference)		1 (reference)		1 (reference)	
1-4	0.82 (0.79, 0.86)	<.001	0.96 (0.92, 1.00)	0.057	0.93 (0.89, 0.97)	<.001
≥ 5	1.80 (1.62, 1.99)	<.001	1.21 (1.09, 1.34)	<.001	1.10 (0.99, 1.22)	0.081
Regular physical activity (days/week)						
None	1 (reference)		1 (reference)		1 (reference)	
1-4	0.88 (s, 0.92)	<.001	0.90 (0.86, 0.93)	<.001	0.90 (0.87, 0.94)	<.001
≥ 5	1.35 (1.26, 1.44)	<.001	0.98 (0.92, 1.05)	0.607	0.99 (0.93, 1.06)	0.809
Comorbidities						
Hypertension	2.90 (2.79, 3.01)	<.001	1.38 (1.32, 1.44)	<.001	1.36 (1.30, 1.42)	<.001
Diabetes mellitus	2.51 (2.40, 2.62)	<.001	1.19 (1.13, 1.25)	<.001	1.16 (1.11, 1.22)	<.001
Dyslipidemia	2.07 (2.00, 2.15)	<.001	1.16 (1.11, 1.21)	<.001	1.14 (1.09, 1.19)	<.001

Stroke	3.72 (3.37, 4.11)	<.001	1.22 (1.10, 1.35)	<.001	1.23 (1.11, 1.36)	<.001
Atrial fibrillation	4.71 (4.09, 5.42)	<.001	1.90 (1.65, 2.19)	<.001	1.89 (1.64, 2.18)	<.001
Renal disease	3.32 (3.02, 3.66)	<.001	1.34 (1.22, 1.48)	<.001	1.36 (1.23, 1.50)	<.001
Cancer	2.79 (2.58, 3.01)	<.001	1.58 (1.46, 1.71)	<.001	1.58 (1.46, 1.71)	<.001
Antiphospholipid syndrome	2.65 (2.19, 3.19)	<.001	1.41 (1.16, 1.70)	0.001	1.40 (1.15, 1.69)	0.001
Osteoporotic fracture	1.83 (1.64, 2.06)	<.001	0.92 (0.82, 1.04)	0.169	0.92 (0.82, 1.04)	0.166
Aspartate Aminotransferase (AST) (U/L)	1.04 (1.01, 1.07)	<.001	1.03 (1.01, 1.05)	<.001	1.02 (1.01, 1.04)	<.001
Alanine Aminotransferase (ALT) (U/L)	1.04 (1.01, 1.06)	<.001	1.03 (1.01, 1.04)	<.001	1.03 (1.01, 1.04)	<.001
Mean GGT (U/L)	1.00 (1.00, 1.00)	<.001			1.00 (1.00, 1.00)	<.001
GGT variability with CV						
Q1	1 (reference)		1 (reference)		1 (reference)	
Q2	1.00 (0.95, 1.06)	0.905	1.01 (0.96, 1.07)	0.730	1.00 (0.95, 1.06)	0.937
Q3	1.08 (1.03, 1.14)	0.004	1.06 (1.00, 1.11)	0.044	1.04 (0.98, 1.09)	0.192
Q4	1.32 (1.25, 1.39)	<.001	1.15 (1.09, 1.21)	<.001	1.08 (1.03, 1.14)	0.004

Multivariable model (1) was adjusted for sex, age, body mass index, income levels, smoking, alcohol consumption, regular physical activity, hypertension, diabetes mellitus, dyslipidemia, stroke, atrial fibrillation, renal disease, cancer, antiphospholipid syndrome, osteoporotic fracture, aspartate aminotransferase, and alanine aminotransferase.

Multivariable model (2) was adjusted for sex, age, body mass index, income levels, smoking, alcohol consumption, regular physical activity, hypertension, diabetes mellitus, dyslipidemia, stroke, atrial fibrillation, renal disease, cancer, antiphospholipid syndrome, osteoporotic fracture, aspartate aminotransferase, alanine aminotransferase, and mean GGT.

GGT: Gamma-glutamyl Transferase, HR: hazard ratio, CI: confidence interval, Q: quartile, CV: coefficient of variation, SD: standard deviation, VIM: variability independent of the mean.

Supplementary Table 2. The risk for occurrence of venous thrombosis according to deciles of GGT variability

	Number of participants	Number of events	Event rate (%) (95% CI)	Person-years	Incidence rate (per 1000 person-years)	Multivariable model (1) Adjusted HR (95% CI)	p-value	p-value for trend	Multivariable model (2) Adjusted HR (95% CI)	p-value	p-value for trend
CV								<.001			0.032
D1	108510	1079	0.99 (0.94, 1.05)	1326529.57	0.81	1 (reference)			1 (reference)		
D2	108496	1074	0.99 (0.93, 1.05)	1328459.74	0.81	1.05 (0.96, 1.14)	0.270		1.05 (0.96, 1.14)	0.284	
D3	108526	1031	0.95 (0.89, 1.01)	1328313.60	0.78	1.01 (0.93, 1.10)	0.861		1.00 (0.92, 1.09)	0.932	
D4	108510	1124	1.04 (0.98, 1.10)	1327629.13	0.85	1.08 (1.00, 1.18)	0.063		1.08 (0.99, 1.17)	0.091	
D5	108510	1069	0.99 (0.93, 1.04)	1327248.71	0.81	1.03 (0.94, 1.12)	0.574		1.01 (0.93, 1.10)	0.761	
D6	108511	1107	1.02 (0.96, 1.08)	1325623.00	0.84	1.05 (0.97, 1.15)	0.218		1.04 (0.96, 1.13)	0.380	
D7	108527	1165	1.07 (1.01, 1.14)	1324261.77	0.88	1.09 (1.00, 1.19)	0.040		1.07 (0.98, 1.16)	0.123	
D8	108495	1231	1.13 (1.07, 1.20)	1322022.71	0.93	1.12 (1.03, 1.22)	0.006		1.09 (1.00, 1.18)	0.042	
D9	108510	1310	1.21 (1.14, 1.27)	1317821.12	0.99	1.15 (1.06, 1.25)	0.001		1.10 (1.01, 1.19)	0.023	
D10	108510	1579	1.46 (1.38, 1.53)	1302188.32	1.21	1.24 (1.15, 1.34)	<.001		1.13 (1.05, 1.23)	0.002	
SD								<.001			0.041
D1	108940	955	0.88 (0.82, 0.93)	1337075.01	0.71	1 (reference)			1 (reference)		
D2	108489	955	0.88 (0.82, 0.94)	1331253.62	0.72	0.99 (0.90, 1.08)	0.813		0.99 (0.90, 1.08)	0.800	
D3	108062	1008	0.93 (0.88, 0.99)	1324291.73	0.76	0.99 (0.91, 1.09)	0.892		0.99 (0.91, 1.08)	0.841	
D4	108817	1055	0.97 (0.91, 1.03)	1331586.68	0.79	0.99 (0.91, 1.09)	0.875		0.99 (0.90, 1.08)	0.768	
D5	108059	1136	1.05 (0.99, 1.11)	1320969.26	0.86	1.05 (0.96, 1.15)	0.282		1.04 (0.95, 1.13)	0.405	
D6	108608	1165	1.07 (1.01, 1.13)	1326411.06	0.88	1.05 (0.97, 1.15)	0.241		1.04 (0.95, 1.13)	0.438	
D7	108601	1224	1.13 (1.06, 1.19)	1323991.43	0.92	1.08 (0.99, 1.18)	0.078		1.05 (0.96, 1.15)	0.263	
D8	108504	1245	1.15 (1.08, 1.21)	1320314.82	0.94	1.09 (1.00, 1.19)	0.056		1.04 (0.95, 1.14)	0.370	
D9	108515	1340	1.23 (1.17, 1.30)	1317745.19	1.02	1.17 (1.07, 1.27)	0.001		1.08 (0.99, 1.18)	0.084	
D10	108510	1686	1.55 (1.48, 1.63)	1296458.86	1.30	1.35 (1.24, 1.48)	<.001		1.10 (1.00, 1.21)	0.058	
VIM								0.001			0.039

D1	108507	1135	1.05 (0.99, 1.11)	1324501.85	0.86	1 (reference)		1 (reference)	
D2	108403	1068	0.99 (0.93, 1.04)	1326150.96	0.81	1.01 (0.93, 1.10)	0.866	1.01 (0.93, 1.10)	0.867
D3	108621	1107	1.02 (0.96, 1.08)	1328366.43	0.83	1.05 (0.97, 1.14)	0.240	1.05 (0.97, 1.14)	0.247
D4	108516	1131	1.04 (0.98, 1.10)	1326735.67	0.85	1.07 (0.99, 1.16)	0.103	1.07 (0.98, 1.16)	0.120
D5	108509	1087	1.00 (0.94, 1.06)	1325997.03	0.82	1.02 (0.94, 1.11)	0.600	1.02 (0.94, 1.11)	0.680
D6	108501	1131	1.04 (0.98, 1.10)	1325679.77	0.85	1.07 (0.98, 1.16)	0.126	1.06 (0.97, 1.15)	0.188
D7	108515	1204	1.11 (1.05, 1.17)	1323822.09	0.91	1.12 (1.03, 1.21)	0.008	1.10 (1.02, 1.20)	0.017
D8	108513	1179	1.09 (1.02, 1.15)	1321923.74	0.89	1.08 (1.00, 1.18)	0.053	1.07 (0.99, 1.16)	0.104
D9	108512	1243	1.15 (1.08, 1.21)	1318998.62	0.94	1.09 (1.01, 1.18)	0.036	1.07 (0.99, 1.16)	0.109
D10	108508	1484	1.37 (1.30, 1.44)	1307921.50	1.14	1.18 (1.09, 1.28)	<.001	1.14 (1.05, 1.23)	0.001

Multivariable model (1) was adjusted for sex, age, body mass index, income levels, smoking, alcohol consumption, regular physical activity, hypertension, diabetes mellitus, dyslipidemia, stroke, atrial fibrillation, renal disease, cancer, antiphospholipid syndrome, osteoporotic fracture, aspartate aminotransferase, and alanine aminotransferase.
Multivariable model (2) was adjusted for sex, age, body mass index, income levels, smoking, alcohol consumption, regular physical activity, hypertension, diabetes mellitus, dyslipidemia, stroke, atrial fibrillation, renal disease, cancer, antiphospholipid syndrome, osteoporotic fracture, aspartate aminotransferase, alanine aminotransferase, and mean GGT.
GGT: Gamma-glutamyl Transferase, HR: hazard ratio, CI: confidence interval, Q: quartile, CV: coefficient of variation, SD: standard deviation, VIM: variability independent of the mean.

Supplementary Table 3. Risk factors for the occurrence of venous thrombosis (landmark analysis)

Variable	Crude HR (95% CI)	p-value	Multivariable model (1) Adjusted HR (95% CI)	p-value	Multivariable model (2) Adjusted HR (95% CI)	p-value
Age, years	1.08 (1.07, 1.08)	<.001	1.06 (1.06, 1.07)	<.001	1.06 (1.06, 1.07)	<.001
Sex						
Male	1 (reference)		1 (reference)		1 (reference)	
Female	1.17 (1.13, 1.22)	<.001	1.01 (0.96, 1.06)	0.768	1.06 (1.00, 1.11)	0.040
Body mass index (kg/m2)	1.07 (1.06, 1.07)	<.001	1.04 (1.04, 1.05)	<.001	1.04 (1.03, 1.05)	<.001
Household income						
Q1, lowest	1 (reference)		1 (reference)		1 (reference)	
Q2	0.60 (0.57, 0.63)	<.001	1.01 (0.96, 1.06)	0.796	1.01 (0.96, 1.06)	0.792
Q3	0.49 (0.47, 0.52)	<.001	0.89 (0.84, 0.94)	<.001	0.89 (0.84, 0.94)	<.001
Q4, highest	0.52 (0.49, 0.55)	<.001	0.72 (0.68, 0.77)	<.001	0.72 (0.68, 0.77)	<.001
Smoking status						
Never	1 (reference)		1 (reference)		1 (reference)	
Former	0.87 (0.83, 0.92)	<.001	1.01 (0.95, 1.07)	0.837	1.01 (0.95, 1.07)	0.844
Current	0.78 (0.75, 0.81)	<.001	1.10 (1.05, 1.16)	<.001	1.08 (1.03, 1.14)	0.001
Alcohol consumption (days/week)						
None	1 (reference)		1 (reference)		1 (reference)	
1-4	0.83 (0.79, 0.86)	<.001	0.96 (0.92, 1.00)	0.077	0.93 (0.89, 0.97)	0.001
≥ 5	1.82 (1.64, 2.01)	<.001	1.22 (1.10, 1.36)	<.001	1.11 (1.00, 1.23)	0.055
Regular physical activity (days/week)						
None	1 (reference)		1 (reference)		1 (reference)	
1-4	0.89 (0.85, 0.92)	<.001	0.90 (0.87, 0.94)	<.001	0.90 (0.87, 0.94)	<.001
≥ 5	1.35 (1.26, 1.45)	<.001	0.99 (0.92, 1.06)	0.671	1.00 (0.93, 1.07)	0.877
Comorbidities						
Hypertension	2.90 (2.79, 3.00)	<.001	1.38 (1.32, 1.44)	<.001	1.36 (1.30, 1.42)	<.001
Diabetes mellitus	2.50 (2.39, 2.62)	<.001	1.19 (1.13, 1.25)	<.001	1.16 (1.10, 1.22)	<.001
Dyslipidemia	2.07 (1.99, 2.15)	<.001	1.16 (1.11, 1.21)	<.001	1.14 (1.09, 1.19)	<.001
Stroke	3.69 (3.34, 4.08)	<.001	1.21 (1.09, 1.34)	<.001	1.22 (1.11, 1.36)	<.001
Atrial fibrillation	4.66 (4.05, 5.37)	<.001	1.89 (1.63, 2.18)	<.001	1.88 (1.63, 2.17)	<.001
Renal disease	3.33 (3.02, 3.66)	<.001	1.35 (1.22, 1.49)	<.001	1.37 (1.24, 1.51)	<.001
Cancer	2.74 (2.53, 2.97)	<.001	1.56 (1.43, 1.69)	<.001	1.56 (1.44, 1.69)	<.001
Antiphospholipid syndrome	2.61 (2.16, 3.16)	<.001	1.40 (1.15, 1.70)	0.001	1.39 (1.14, 1.68)	0.001
Osteoporotic fracture	1.84 (1.64, 2.06)	<.001	0.92 (0.82, 1.04)	0.180	0.92 (0.82, 1.04)	0.177
Mean GGT (U/L)	1.00 (1.00, 1.00)	<.001			1.00 (1.00, 1.00)	<.001
GGT variability with CV						
Q1	1 (reference)		1 (reference)		1 (reference)	
Q2	1.00 (0.95, 1.05)	0.952	1.01 (0.95, 1.06)	0.870	1.00 (0.95, 1.05)	0.919
Q3	1.08 (1.02, 1.14)	0.006	1.05 (1.00, 1.11)	0.060	1.03 (0.98, 1.09)	0.242
Q4	1.31 (1.25, 1.38)	<.001	1.14 (1.09, 1.20)	<.001	1.08 (1.02, 1.13)	0.007

Multivariable model (1) was adjusted for sex, age, body mass index, income levels, smoking, alcohol consumption, regular physical activity, hypertension, diabetes mellitus, dyslipidemia, stroke, atrial fibrillation, renal disease, cancer, antiphospholipid syndrome, osteoporotic fracture, aspartate aminotransferase, and alanine aminotransferase.

Multivariable model (2) was adjusted for sex, age, body mass index, income levels, smoking, alcohol consumption, regular physical activity, hypertension, diabetes mellitus, dyslipidemia, stroke, atrial fibrillation, renal disease, cancer, antiphospholipid syndrome, osteoporotic fracture, aspartate aminotransferase, alanine aminotransferase, and mean GGT.

HR: hazard ratio, CI: confidence interval, Q: quartile, GGT: Gamma-glutamyl Transferase, CV: coefficient of variation

Supplementary Table 4. The risk for occurrence of venous thrombosis according to quartiles of GGT variability (landmark analysis)

						Multivariable model (1)			Multivariable model (2)		
	Number of participants	Number of events	Event rate (%) (95% CI)	Person-years	Incidence rate (per 1000 person-years)	Adjusted HR (95% CI)	p-value	p-value for trend	Adjusted HR (95% CI)	p-value	p-value for trend
CV								<.001			0.014
Q1	271327	2654	0.98 (0.94, 1.02)	3320263.34	0.80	1 (reference)			1 (reference)		
Q2	271225	2652	0.98 (0.94, 1.02)	3318712.57	0.80	1.01 (0.95, 1.06)	0.870		1.00 (0.95, 1.05)	0.919	
Q3	271281	2850	1.05 (1.01, 1.09)	3311379.82	0.86	1.05 (1.00, 1.11)	0.060		1.03 (0.98, 1.09)	0.242	
Q4	271272	3446	1.27 (1.23, 1.31)	3281470.35	1.05	1.14 (1.09, 1.20)	<.001		1.08 (1.02, 1.13)	0.007	
SD								<.001			0.018
Q1	271920	2404	0.88 (0.85, 0.92)	3336659.73	0.72	1 (reference)			1 (reference)		
Q2	270447	2638	0.98 (0.94, 1.01)	3309285.73	0.80	1.01 (0.95, 1.06)	0.870		1.00 (0.94, 1.06)	0.966	
Q3	271459	2920	1.08 (1.04, 1.11)	3311524.86	0.88	1.05 (0.99, 1.11)	0.107		1.02 (0.97, 1.08)	0.433	
Q4	271279	3640	1.34 (1.30, 1.39)	3274355.76	1.11	1.23 (1.16, 1.31)	<.001		1.09 (1.02, 1.16)	0.008	
VIM								0.001			0.027
Q1	271281	2730	1.01 (0.97, 1.04)	3315799.82	0.82	1 (reference)			1 (reference)		
Q2	271275	2729	1.01 (0.97, 1.04)	3316713.91	0.82	1.03 (0.98, 1.09)	0.299		1.03 (0.97, 1.08)	0.362	
Q3	271276	2897	1.07 (1.03, 1.11)	3310783.49	0.88	1.08 (1.02, 1.14)	0.005		1.07 (1.01, 1.13)	0.014	
Q4	271273	3246	1.20 (1.16, 1.24)	3288528.86	0.99	1.10 (1.04, 1.16)	<.001		1.07 (1.02, 1.13)	0.010	

Multivariable model (1) was adjusted for sex, age, body mass index, income levels, smoking, alcohol consumption, regular physical activity, hypertension, diabetes mellitus, dyslipidemia, stroke, atrial fibrillation, renal disease, cancer, antiphospholipid syndrome, osteoporotic fracture, aspartate aminotransferase, and alanine aminotransferase.
Multivariable model (2) was adjusted for sex, age, body mass index, income levels, smoking, alcohol consumption, regular physical activity, hypertension, diabetes mellitus, dyslipidemia, stroke, atrial fibrillation, renal disease, cancer, antiphospholipid syndrome, osteoporotic fracture, aspartate aminotransferase, alanine aminotransferase, and mean GGT.
GGT: Gamma-glutamyl Transferase, CI: confidence interval, HR: hazard ratio, CV: coefficient of variation, Q: quartile, SD: standard deviation, VIM: variability independent of the mean.

Supplementary Table 5. The risk for occurrence of deep vein thrombosis according to quartiles of GGT variability

	Number of participants	Number of events	Event rate (%) (95% CI)	Person-years	Incidence rate (per 1000 person-years)	Adjusted HR (95% CI)	p-value	p-value for trend
CV								0.005
Q1	271327	1099	0.41 (0.38, 0.43)	3326366.96	0.33			
Q2	271225	1062	0.39 (0.37, 0.42)	3324697.33	0.32	0.98 (0.90, 1.07)	0.626	
Q3	271281	1194	0.44 (0.42, 0.47)	3317824.30	0.36	1.08 (1.00, 1.17)	0.066	
Q4	271272	1355	0.50 (0.47, 0.53)	3289208.39	0.41	1.11 (1.03, 1.21)	0.009	
SD								<.001
Q1	271920	1097	0.40 (0.38, 0.43)	3341619.66	0.33			
Q2	270447	1085	0.40 (0.38, 0.43)	3315405.42	0.33	0.95 (0.87, 1.03)	0.213	
Q3	271459	1122	0.41 (0.39, 0.44)	3318450.91	0.34	0.95 (0.87, 1.04)	0.294	
Q4	271279	1406	0.52 (0.49, 0.55)	3282621.00	0.43	1.16 (1.06, 1.27)	0.001	
VIM								0.027
Q1	271281	1108	0.41 (0.38, 0.43)	3322194.69	0.33			
Q2	271275	1085	0.40 (0.38, 0.42)	3323029.39	0.33	1.01 (0.93, 1.10)	0.852	
Q3	271276	1198	0.44 (0.42, 0.47)	3317168.54	0.36	1.10 (1.01, 1.19)	0.024	
Q4	271273	1319	0.49 (0.46, 0.51)	3295704.36	0.40	1.10 (1.01, 1.19)	0.026	

Multivariable model was adjusted for sex, age, body mass index, income levels, smoking, alcohol consumption, regular physical activity, hypertension, diabetes mellitus, dyslipidemia, stroke, atrial fibrillation, renal disease, cancer, antiphospholipid syndrome, osteoporotic fracture, aspartate aminotransferase, alanine aminotransferase, and mean GGT.

GGT: Gamma-glutamyl Transferase, CI: confidence interval, HR: hazard ratio, CV: coefficient of variation, Q: quartile, SD: standard deviation, VIM: variability independent of the mean.

Supplementary Table 6. The risk for occurrence of pulmonary thromboembolism according to quartiles of GGT variability

	Number of participants	Number of events	Event rate (%) (95% CI)	Person-years	Incidence rate (per 1000 person-years)	Adjusted HR (95% CI)	p-value	p-value for trend
CV								<.001
Q1	271327	738	0.27 (0.25, 0.29)	3328661.66	0.22			
Q2	271225	679	0.25 (0.23, 0.27)	3326991.87	0.20	0.94 (0.85, 1.05)	0.265	
Q3	271281	746	0.27 (0.26, 0.29)	3320321.49	0.23	1.02 (0.92, 1.13)	0.704	
Q4	271272	946	0.35 (0.33, 0.37)	3291385.56	0.29	1.17 (1.06, 1.29)	0.002	
SD								<.001
Q1	271920	647	0.24 (0.22, 0.26)	3344209.90	0.19			
Q2	270447	701	0.26 (0.24, 0.28)	3317615.57	0.21	0.98 (0.88, 1.09)	0.709	
Q3	271459	783	0.29 (0.27, 0.31)	3320525.10	0.24	1.05 (0.94, 1.17)	0.392	
Q4	271279	978	0.36 (0.34, 0.38)	3285009.99	0.30	1.27 (1.14, 1.42)	<.001	
VIM								0.010
Q1	271281	744	0.27 (0.25, 0.29)	3324493.20	0.22			
Q2	271275	706	0.26 (0.24, 0.28)	3325221.99	0.21	1.00 (0.90, 1.11)	0.955	
Q3	271276	761	0.28 (0.26, 0.30)	3319712.34	0.23	1.07 (0.97, 1.18)	0.189	
Q4	271273	898	0.33 (0.31, 0.35)	3297933.03	0.27	1.15 (1.05, 1.27)	0.005	

Multivariable model was adjusted for sex, age, body mass index, income levels, smoking, alcohol consumption, regular physical activity, hypertension, diabetes mellitus, dyslipidemia, stroke, atrial fibrillation, renal disease, cancer, antiphospholipid syndrome, osteoporotic fracture, aspartate aminotransferase, alanine aminotransferase, and mean GGT.

GGT: Gamma-glutamyl Transferase, CI: confidence interval, HR: hazard ratio, CV: coefficient of variation, Q: quartile, SD: standard deviation, VIM: variability independent of the mean.

Supplementary Table 7. The risk for occurrence of intraabdominal thrombosis according to quartiles of GGT variability

	Number of participants	Number of events	Event rate (%) (95% CI)	Person-years	Incidence rate (per 1000 person-years)	Adjusted HR (95% CI)	p-value	p-value for trend
CV								<.001
Q1	271327	1166	0.43 (0.41, 0.45)	3324776.47	0.35			
Q2	271225	1218	0.45 (0.42, 0.47)	3323078.13	0.37	1.03 (0.95, 1.12)	0.437	
Q3	271281	1268	0.47 (0.44, 0.49)	3316141.45	0.38	1.04 (0.96, 1.12)	0.399	
Q4	271272	1562	0.58 (0.55, 0.60)	3286603.80	0.48	1.13 (1.05, 1.22)	0.002	
SD								<.001
Q1	271920	968	0.36 (0.33, 0.38)	3341137.35	0.29			
Q2	270447	1180	0.44 (0.41, 0.46)	3313785.26	0.36	1.07 (0.99, 1.17)	0.105	
Q3	271459	1370	0.50 (0.48, 0.53)	3316090.43	0.41	1.13 (1.04, 1.23)	0.006	
Q4	271279	1696	0.63 (0.60, 0.65)	3279586.81	0.52	1.27 (1.16, 1.38)	<.001	
VIM								0.048
Q1	271281	1237	0.46 (0.43, 0.48)	3320312.89	0.37			
Q2	271275	1249	0.46 (0.43, 0.49)	3321162.93	0.38	1.03 (0.95, 1.11)	0.511	
Q3	271276	1304	0.48 (0.45, 0.51)	3315595.87	0.39	1.06 (0.98, 1.14)	0.171	
Q4	271273	1424	0.52 (0.50, 0.55)	3293528.17	0.43	1.08 (1.01, 1.14)	0.035	

Multivariable model was adjusted for sex, age, body mass index, income levels, smoking, alcohol consumption, regular physical activity, hypertension, diabetes mellitus, dyslipidemia, stroke, atrial fibrillation, renal disease, cancer, antiphospholipid syndrome, osteoporotic fracture, aspartate aminotransferase, alanine aminotransferase, and mean GGT.

GGT: Gamma-glutamyl Transferase, CI: confidence interval, HR: hazard ratio, CV: coefficient of variation, Q: quartile, SD: standard deviation, VIM: variability independent of the mean.

Supplementary Table 8. The risk for occurrence of other venous thrombosis according to quartiles of GGT variability

	Number of participants	Number of events	Event rate (%) (95% CI)	Person-years	Incidence rate (per 1000 person-years)	Adjusted HR (95% CI)	p-value	p-value for trend
CV								0.037
Q1	271327	1084	0.40 (0.38, 0.42)	3325021.55	0.33			
Q2	271225	1138	0.42 (0.40, 0.44)	3323299.41	0.34	1.04 (0.96, 1.13)	0.383	
Q3	271281	1164	0.43 (0.40, 0.45)	3316462.61	0.35	1.02 (0.94, 1.11)	0.614	
Q4	271272	1407	0.52 (0.49, 0.55)	3286902.52	0.43	1.09 (1.01, 1.19)	0.028	
SD								0.004
Q1	271920	917	0.34 (0.32, 0.36)	3341320.11	0.27			
Q2	270447	1110	0.41 (0.39, 0.43)	3313964.39	0.34	1.07 (0.98, 1.16)	0.161	
Q3	271459	1260	0.46 (0.44, 0.49)	3316391.29	0.38	1.09 (1.00, 1.19)	0.055	
Q4	271279	1506	0.56 (0.53, 0.58)	3280010.29	0.46	1.18 (1.08, 1.29)	<.001	
VIM								0.039
Q1	271281	1138	0.42 (0.40, 0.44)	3320628.28	0.34			
Q2	271275	1160	0.43 (0.40, 0.45)	3321375.93	0.35	1.04 (0.96, 1.13)	0.387	
Q3	271276	1201	0.44 (0.42, 0.47)	3315864.00	0.36	1.06 (0.97, 1.15)	0.184	
Q4	271273	1294	0.48 (0.45, 0.50)	3293817.87	0.39	1.07 (1.01, 1.12)	0.036	

Multivariable model was adjusted for sex, age, body mass index, income levels, smoking, alcohol consumption, regular physical activity, hypertension, diabetes mellitus, dyslipidemia, stroke, atrial fibrillation, renal disease, cancer, antiphospholipid syndrome, osteoporotic fracture, aspartate aminotransferase, alanine aminotransferase, and mean GGT.

GGT: Gamma-glutamyl Transferase, CI: confidence interval, HR: hazard ratio, CV: coefficient of variation, Q: quartile, SD: standard deviation, VIM: variability independent of the mean.

Supplementary Figure 1. Kaplan-Meier survival curves for occurrence of deep VT according to GGT variability.

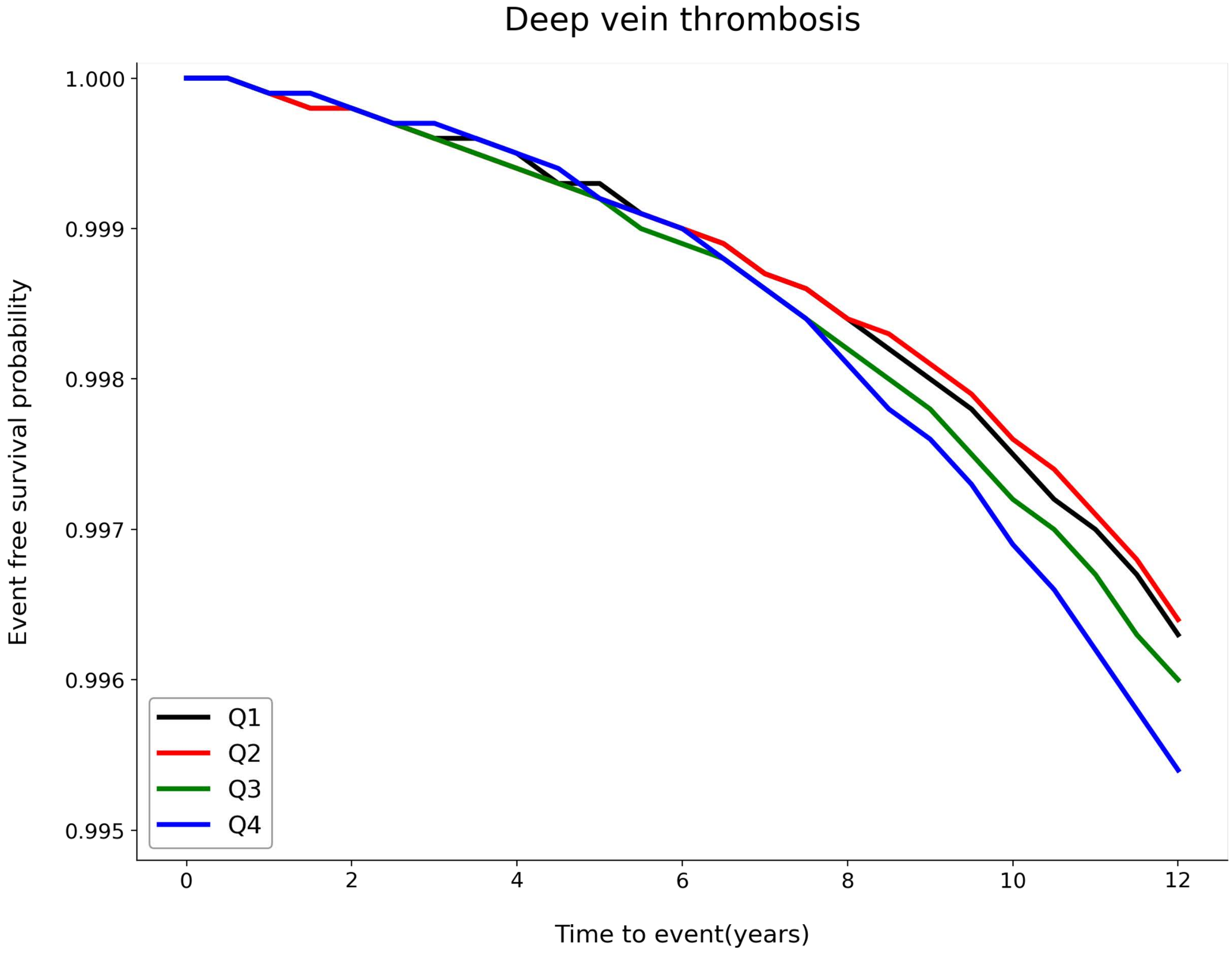

Q1	271327	270709	269776	268649	267336	265814	262880
Q2	271225	270618	269681	268505	267209	265673	262794
Q3	271281	270545	269443	268178	266643	264877	261564
Q4	271272	269973	268227	266221	263927	261408	256980

Supplementary Figure 2. Kaplan-Meier survival curves for occurrence of pulmonary thromboembolism according to GGT variability.

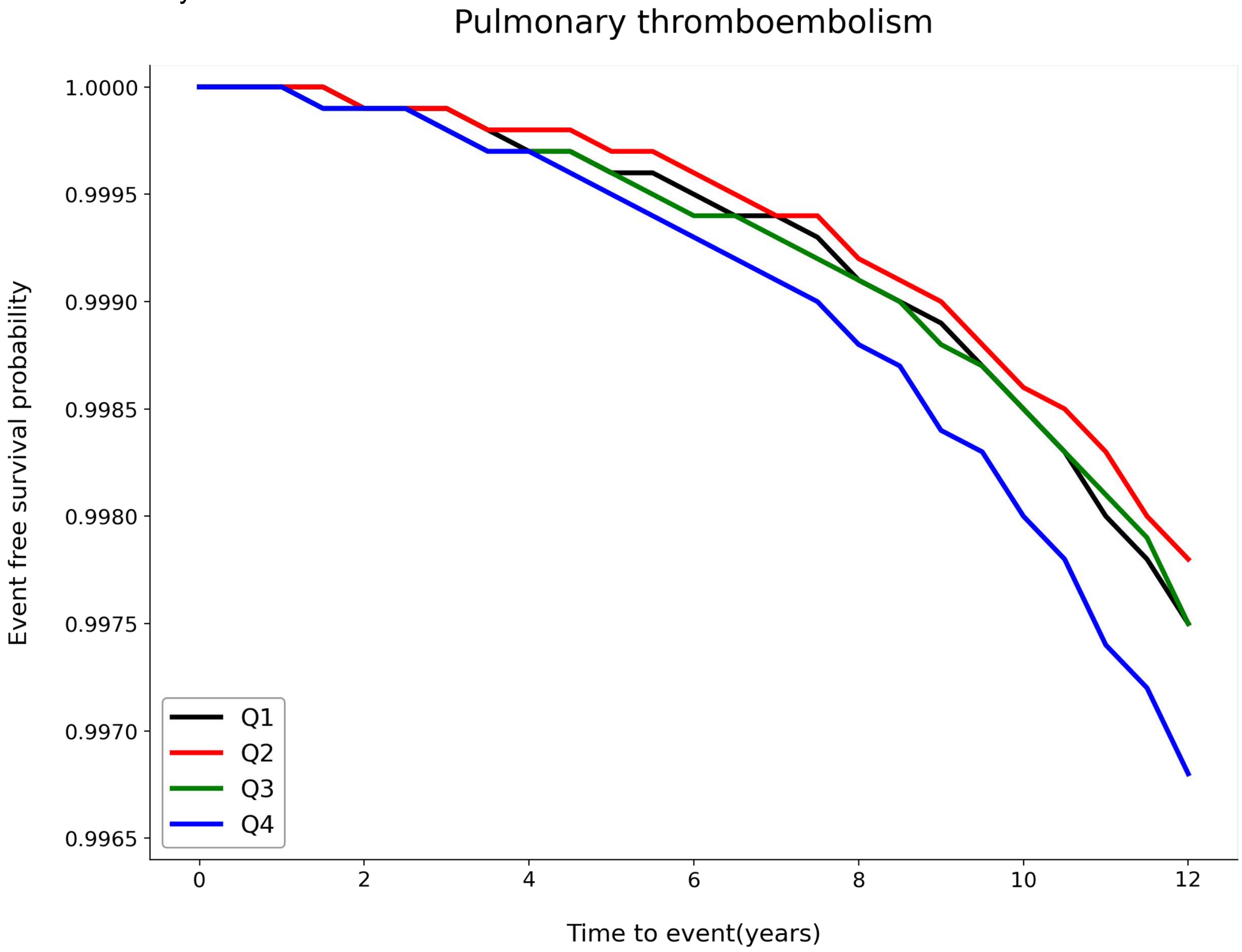

Q1	271327	270760	269860	268803	267566	266141	263294
Q2	271225	270665	269795	268671	267434	265972	263207
Q3	271281	270575	269528	268341	266909	265259	262026
Q4	271272	270007	268300	266349	264144	261738	257428

Supplementary Figure 3. Kaplan-Meier survival curves for occurrence of intraabdominal thrombosis according to GGT variability.

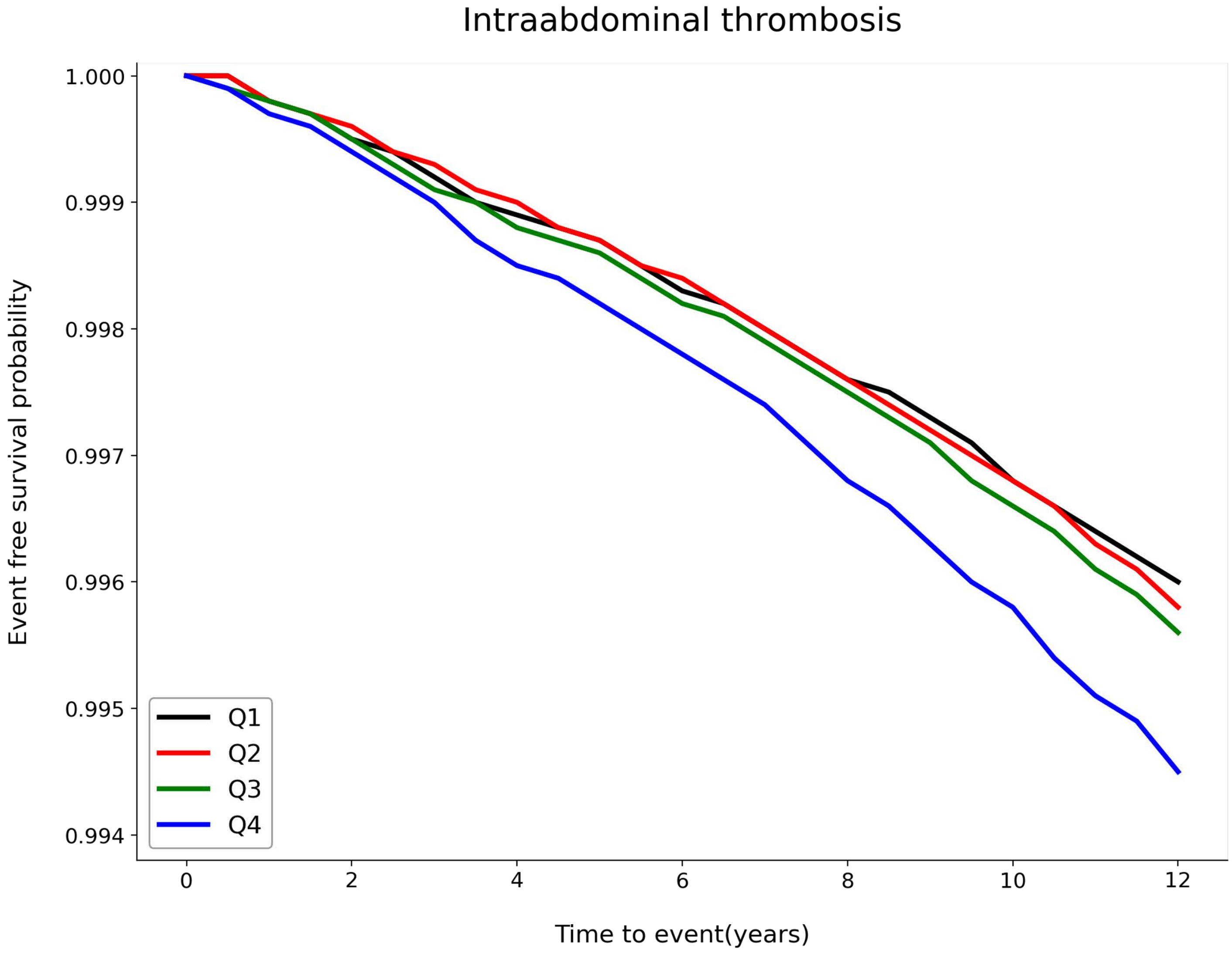

Q1	271327	270648	269630	268479	267141	265654	262788
Q2	271225	270568	269569	268346	267018	265458	262666
Q3	271281	270461	269278	267998	266461	264718	261452
Q4	271272	269869	267991	265937	263628	261153	256815

Supplementary Figure 4. Kaplan-Meier survival curves for occurrence of other VT according to GGT variability.

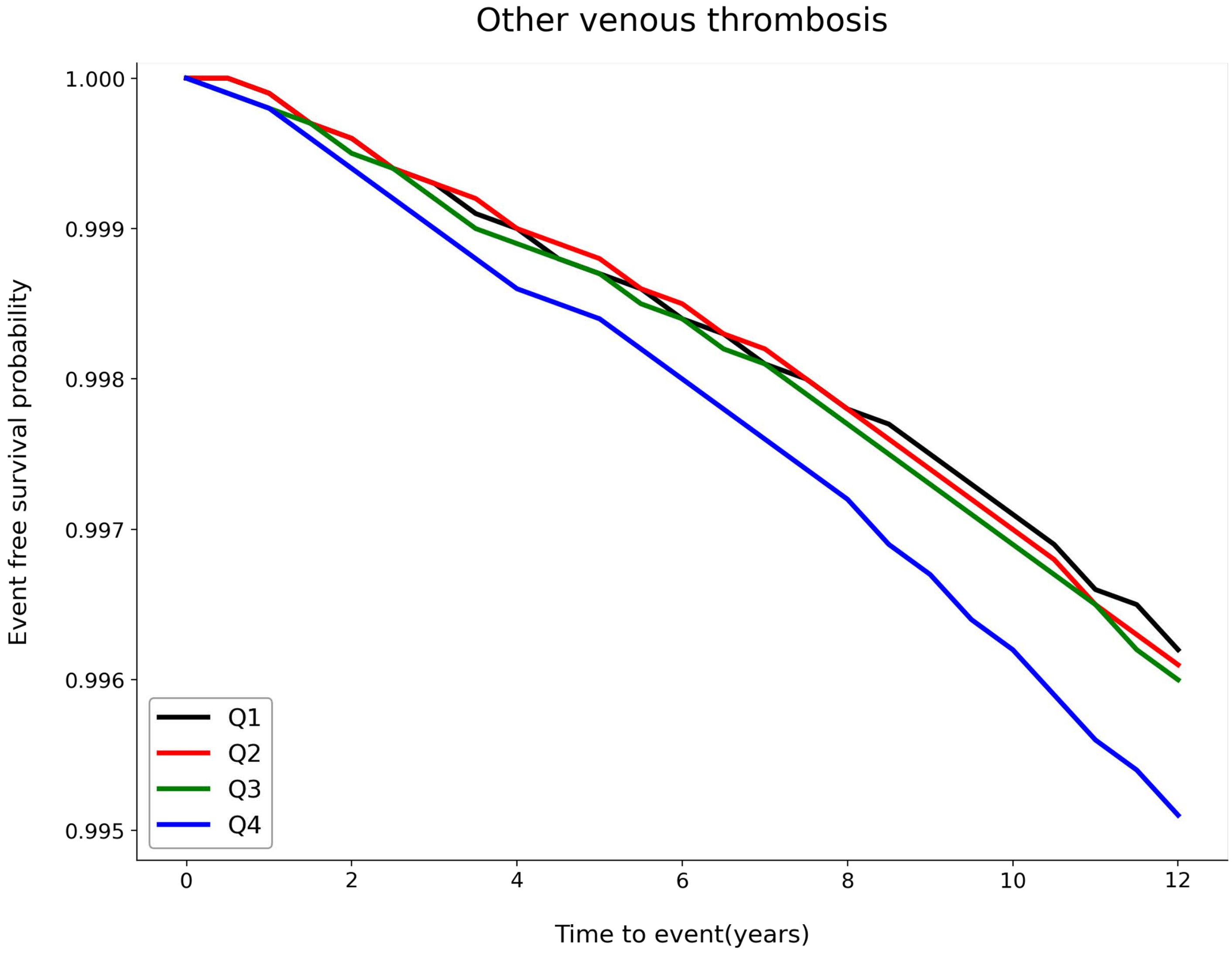

Q1	271327	270652	269638	268496	267169	265691	262834
Q2	271225	270571	269574	268360	267040	265492	262710
Q3	271281	270465	269291	268020	266496	264768	261516
Q4	271272	269874	268001	265955	263659	261196	256878
